# Non-Local Sensitivity Analysis and Numerical Homogenization in Optimal Design of Single-Wall Corrugated Board Packaging

**DOI:** 10.3390/ma15030720

**Published:** 2022-01-18

**Authors:** Damian Mrówczyński, Anna Knitter-Piątkowska, Tomasz Garbowski

**Affiliations:** 1 Doctoral School, Department of Biosystems Engineering, Poznan University of Life Sciences, Wojska Polskiego 28, 60-637 Poznan, Poland; damian.mrowczynski@up.poznan.pl; 2 Institute of Structural Analysis, Poznan University of Technology, Piotrowo 5, 60-965 Poznan, Poland; anna.knitter-piatkowska@put.poznan.pl; 3 Department of Biosystems Engineering, Poznan University of Life Sciences, Wojska Polskiego 50, 60-627 Poznan, Poland

**Keywords:** corrugated cardboard, non-local sensitivity analysis, numerical homogenization, optimal packaging, box compressive strength, critical load, orthotropic plate

## Abstract

The optimal selection of the composition of corrugated cardboard dedicated to specific packaging structures is not an easy task. The use of lighter boards saves material, but at the same time increases the risk of not meeting the guaranteed load capacity. Therefore, the answer to the question “in which layer the basis weight of the paper should be increased?” is not simple or obvious. The method proposed here makes it easy to understand which components and to what extent they affect the load-bearing capacity of packages of various dimensions. The use of numerical homogenization allows for a quick transformation of a cardboard sample, i.e., a representative volume element (RVE) into a flat plate structure with effective parameters describing the membrane and bending stiffness. On the other hand, the use of non-local sensitivity analysis makes it possible to find the relationship between the parameters of the paper and the load capacity of the packaging. The analytical procedures presented in our previous studies were used here to determine (1) the edge crush resistance, (2) critical load, and (3) the load capacity of corrugated cardboard packaging. The method proposed here allows for obtaining a comprehensive and hierarchical list of the parameters that play the most important role in the process of optimal packaging design.

## 1. Introduction

The growing ecological awareness and concern for the natural environment with the simultaneous, inevitable increase in the needs for the purchase, production, transport, and storage of diverse products, have forced the pursuit for environmentally friendly solutions, including easy to dispose of and, more importantly, recyclable packaging. At this point, it should be emphasized that corrugated cardboard boxes fit perfectly into this trend. The merits of such a package include also the simplicity of shaping through appropriately designed creasing, easy formation of openings, ventilation holes and perforations, and convenient color printing, which attracts the vendee in the case of shelf-ready packaging (SRP) or retail-ready packaging (RRP).

The high demand on the market has caused the intensive development of a distinct branch of industry, i.e., packaging production, and thus the rapid progress of scientific research in this field. The issue of strength evaluation of corrugated cardboard products is the subject of continuous, extensive studies. The corrugated cardboard consists of layers, therefore their proper selection and combination determine its relevant load-bearing capacity. Two characteristic in-plane directions of orthotropy indissolubly connect to the mechanical strength of the paperboard, namely the machine direction (MD), which is perpendicular to the main axis of the fluting and parallel to the paperboard fiber alignment, and cross direction (CD), which is parallel to the fluting.

The optimal selection of the composition of corrugated cardboard layers is crucial for the load-bearing capacity of packages. There is a possibility of the application of lighter boards as well as kraft or recycled papers (with reduced mechanical parameters), which saves material, thereby reducing cost. However, the risk of not complying with the guaranteed load capacity cannot be ignored. Another aspect that has to be taken into account is the behavior of the cardboard boxes strictly connected with their dimensions. In the case of high packaging, the buckling strength is important, while in low and stocky boxes, a high edge crush strength is significant. The method discussed in this paper allows for estimating which components and to what extent they affect the load-bearing capacity of packages of various dimensions.

There are plenty of approaches for assessment of the cardboard load-bearing capacity that can be found in the literature. Generally, they are classified as analytical, numerical, and experimental methods. Compressive, tensile, or bursting strength tests are fundamental physical examinations to assess the load-bearing capacity of corrugated cardboard. The box compression test (BCT), bending stiffness (BS) and the edge crush test (ECT) are the most widespread [1,2,3,4]. Starting from the early 1950s, analytical methods have been proposed [5,6,7], where, in the formulae, three groups of parameters, such as paper, board, and box parameters, can be distinguished [8]. In the first set, the ring crush test (RCT), the Concora liner test (CLT), liner type, weights of liner and fluting, corrugation ratio, and a constant related to fluting are included. In the second one, thickness, flexural stiffnesses in MD and CD, ECT, and moisture content are included. In the latter, the dimensions and perimeter of the box, applied load ratio, stacking time, buckling ratio, and printed ratio are present. The McKee’s analytical formula [7] is widely used in the packaging industry because of its austerity, which leads to quick and easy solutions for practical applications, but only applies to simple standard boxes. This fact was and, in fact, still is an impulse for further development of the method, e.g., through the modification of constants and exponents [9], expanding the range of cutting methods and equipment [10], introducing the dimensions of the box [11] or including the Poisson’s ratio [12]. Further alteration of the above-mentioned approach for solving more complex problems has been presented in [13] and, recently, while taking into consideration the buckling phenomena of the orthotropic cardboards, in [14].

The finite element method (FEM) is a well-known and common numerical approach applied also for determining the strength of the cardboard boxes. FEM simulations of the paperboard creasing in order to obtain its mechanical properties have been presented in, e.g., [15,16,17,18,19,20]. The discussion on the numerical strength estimation of corrugated board packages can be found in [21,22,23,24]. Torsional and transversal stiffness of orthotropic paper materials while involving FEM has been considered in [25,26,27,28,29], bending stiffness has been examined in [30,31], whereas the buckling and post-buckling phenomena have been described in [32]. In [33], FEM was engaged for verification of the stress analysis results of adhesively bonded joints of the corrugated sandwich structure obtained while using the cohesive zone method. The degraded cohesive zone model was also used for modeling the damage propagation in the adhesive layer of aged sandwich corrugated beams [34], and the numerical prediction on structural strength degradation showed a good compliance. Finite element analysis of hot melt adhesive joints in carton board was thoroughly discussed in [35]. While performing the numerical simulations in order to test corrugated cardboard, knowledge of the material properties of each layer is essential. This is a challenging task due to anisotropy of the paper-based materials. The method that allows for facilitating one single layer is called homogenization and consists of determining the equivalent stiffnesses and effective thicknesses of the model. Analytical homogenization is based on the equations of the classical theory of materials’ strength or on the classical theory of laminates [36]. Numerical homogenization, which is the most universal, is based on the finite element method, where first, a numerical model of a representative volume element (RVE) is created [37]. A multiple scales asymptotic homogenization approach can be found in [38], whereas the asymptotic homogenization technique is in [39]. Homogenization of the corrugated board can be carried out in two variants, i.e., homogenization to one layer or homogenization of fluting to the inner layer of the laminate. Such a method has been extensively employed over the last years [40,41,42,43,44,45,46,47,48,49] due to substantial savings in computation time while maintaining the accuracy of the results.

Measurement from an experiment, in other words physical testing, is very common in the paper industry for the estimation of the corrugated board load-bearing capacity. A number of typical tests have been developed to standardize the process of the characterization of corrugated cardboard mechanical properties. Apart from the above-mentioned ECT and BCT, the bending test (BNT), the shear stiffness test (SST), and the torsional stiffness test (TST) are applicable. Bursting and humidity testing are also common. In order to collect the data from the outer surface of the sample during testing, video extensometry can be applied. This technique is based on the measurement of the relative distances between pairs of points traced across images captured at different load values [50,51]. Such a procedure is analogous, but simpler, to the digital image correlation (DIC), which is a full-field non-contact optical measurement procedure. Due to the very high accuracy of data acquisition, it is gaining more recognition in the field of experimental mechanics [29,52,53,54,55,56,57,58].

One cannot forget that there are many factors that diminish the compression strength of corrugated paperboard boxes [59], such as openings, ventilation holes and perforations or indentations [60,61,62,63,64,65], shifted creases on the flaps [66], time and conditions of storage [67,68], and stacking load [47,69,70]. The influence of the box geometry as well as the composition and arrangement of the corrugated board layers on the change of the buckling force, edge crushing (ECT), and the compressive box strength resistance (BCT) are the elements that need to be considered when assessing the load capacity of the box. Another very important factor that needs to be mentioned because of its high impact on the cardboard strength is the profile of a corrugated web, labeled by letters A, B, C, E, and F. The difference between them is in flute height (A type is the tallest, F is the lowest), wavelength, and take-up factor, which is a quantification of the fluting length per unit length of the board [25]. For the most common packages, the cardboard with B and C flutes is applied; for big boxes A flute is applied and for the smallest one, e.g., cosmetic packaging, E and F fluting is applied. Moreover, while speaking of the double-wall corrugated cardboard, different combinations of fluting are applied, e.g., BC, BE, AE, FE, or EB, and vice versa.

A detailed analysis of the sensitivity in the context of observing changes in the value of the global buckling force, ECT, and BCT resulting from minor perturbations of the parameters of the calculation model, allows for answering the questions of many manufacturers—which parameters of the paper/corrugated board have the greatest impact on the load-bearing capacity of the box? In the paper, after in-depth research, the authors present a comprehensive and hierarchical list of parameters that play the most important role in the process of optimal packaging design.

## 2. Materials and Methods

### 2.1. Material Parameters and Corrugated Cardboard Geometry

**Figure 1:**
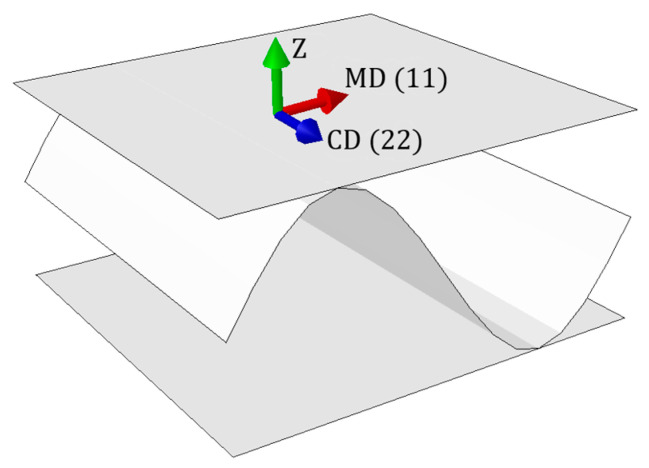
Material orientation. Machine Direction (MD), Cross Direction (CD) and thickness direction (Z).

Corrugated cardboard is a fibrous material, which results in a strong orthotropy. The mechanical properties of the components depend on the orientation of the fibers in the layers. Two main directions can be determined: the machine direction (MD) along the fibers, and the cross direction (CD) across the fibers, perpendicular to the MD. In machine direction, the material is two to three times stiffer in tensile/bending and almost two times the shear/torsion than in the cross direction. The MD is along the waves (see Figure 1), which compensates for the weaker material properties in the cross direction.

The mechanical properties of the layers, such as the moduli of elasticity $E_{1}\;\left(E_{MD}\right)$ and $E_{2}\;\left(E_{CD}\right)$, and the compression strength $SCT_{CD}$, can be determined based on the grammage of the paper from the Mondi technical data [71].

An example of calculating the stiffness modulus $E_{1}$ and $E_{2}$ from the MONDI specifications is as follows (see Table 1):

(1) $$E_{1}=TS_{MD}\frac{grm}{thk},\;\;\;\;\;\;\;\;\;\;\;E_{2}=TS_{CD}\frac{grm}{thk},$$

where grm is grammage ($g/m^{2}$), thk is paper thickness (mm), $TS_{MD}$ is the tensile stiffness index in MD (Nmm/g), and $TS_{CD}$ is the tensile stiffness index in CD. It is assumed that the paper with a grammage of 100 $g/m^{2}$ has a thickness equal to 160 μm.

Poisson’s ratio $\nu_{12}$ can be computed from the empirical formula [72]:

(2) $$\nu_{12}=0.293\sqrt{\frac{E_{2}}{E_{1}}}\;.$$

The in-plane shear stiffness $G_{12}$ is approximated by the formula [72]:

(3) $$G_{12}=0.387\sqrt{E_{1}E_{2}}\;.$$

The transverse shear stiffnesses can be determined from [73] as:

(4) $$G_{13}=\frac{E_{1}}{55}\;,\;\;\;\;\;\;\;\;\;\;\;\;\;\;\;G_{23}=\frac{E_{2}}{35}\;.$$

Wave height, period, and take-up factor are selected based on the wave type [25], as specified in Table 2.

### 2.2. Homogenization Technique

To calculate the BCT value of the packaging, it is necessary to know the stiffness of the corrugated cardboard, which can be obtained using the numerical homogenization method. In the present study, the method based on the elastic energy equivalence between the full RVE model and the simplified shell model was applied. The described method was proposed by Biancolini [37], and was then extended to include transversal shear stiffness by Garbowski and Gajewski [74]. The RVE (representative volume element) is a small, periodic section of the 3D corrugated cardboard structure. All theoretical issues related to the constitutive model can be found in [74]. Only basic information is presented below.

**Figure 2:**
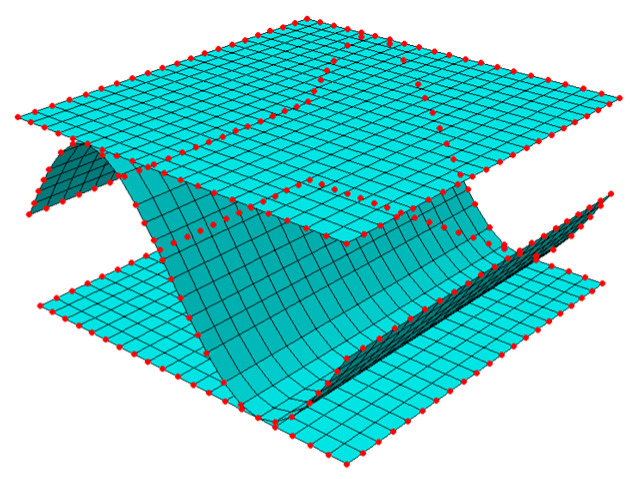
Finite elements and external (red color) and internal nodes of the RVE.

The finite element formulation for a linear analysis can be expressed as follows:

(5) $$K_{e}\;u_{e}=F_{e}\;,$$

where $K_{e}$ is a statically condensed global stiffness matrix of the RVE, $u_{e}$ is a displacement vector, and $F_{e}$ is a vector of the nodal forces. Subscript e means values for external nodes only. In Figure 2, the finite element mesh and mesh nodes of RVE are shown.

Static condensation is the process of removing unknown degrees of freedom (DOF) and leaving behind selected degrees of freedom, called principal DOFs (or primary unknowns). In this case, internal nodes are removed and external nodes are the principal DOFs. The condensed stiffness matrix of the external nodes can be computed from the following formula:

(6) $$K_{e}=K_{ee}-K_{ei}\;K_{ii}{}^{-1}\;K_{ie}\;,$$

where the subarrays are related to the external (subscript *e*) and internal (subscript *i*) nodes:

(7) $$\left[\begin{array}{cc} K_{ee} & K_{ei}\\ K_{ie} & K_{ii} \end{array}\right]\left[\begin{array}{c} u_{e}\\ u_{i} \end{array}\right]=\left[\begin{array}{c} F_{e}\\ 0 \end{array}\right]\;.$$

After static condensation, the total elastic strain energy can be presented as the work of external forces on the corresponding displacements:

(8) $$E=\frac{1}{2}\;u_{e}^{T}\;F_{e}\;.$$

The balance of the total energy between the full RVE model and the simplified shell model is ensured by appropriate definition of displacements of external nodes and taking into account membrane and bending behavior [74]. The generalized displacements are related to the generalized strains on the RVE edges:

(9) $$u_{i}=H_{i}\;\varepsilon_{i}\;,$$

where the $H_{i}$ matrix can be determined for each node ($x_{i}=x$, $y_{i}=y$, $z_{i}=z$):

(10) $${\left[\begin{array}{c} u_{x}\\ u_{y}\\ u_{z}\\ \theta_{x}\\ \theta_{y} \end{array}\right]}_{i}={\left[\begin{array}{cccccccc} x & 0 & y/2 & xz & 0 & yz/2 & z/2 & 0\\ 0 & y & x/2 & 0 & yz & xz/2 & 0 & z/2\\ 0 & 0 & 0 & -x^{2}/2 & -y^{2}/2 & -xy/2 & x/2 & y/2\\ 0 & 0 & 0 & 0 & -y & -x/2 & 0 & 0\\ 0 & 0 & 0 & x & 0 & y/2 & 0 & 0 \end{array}\right]}_{i}{\left[\begin{array}{c} \varepsilon_{x}\\ \varepsilon_{y}\\ \gamma_{xy}\\ \kappa_{x}\\ \kappa_{y}\\ \kappa_{xy}\\ \gamma_{xz}\\ \gamma_{yz} \end{array}\right]}_{i}\;$$

The transformation matrix, $H_{i}$, presented above, links the generalized displacement of each node on the boundary with the generalized strain vector of the corrugated board RVE model. More details on the derivation of such a matrix within the Kirchhoff−Love assumption can be found in [37], while the derivation within the Reissner−Mindlin theory can be found in [74].

Using the definition of the elastic strain energy:

(11) $$E=\frac{1}{2}u_{e}^{T}\;K\;u_{e}=\frac{1}{2}\varepsilon_{e}^{T}\;H_{e}^{T}\;K\;H_{e}\;\varepsilon_{e}$$

and considering a finite element subjected to bending, tension and transverse shear, the elastic internal energy can be represented by the formula:

(12) $$E=\frac{1}{2}\epsilon_{e}^{T}\;H_{k}\;\varepsilon_{e}\left\{area\right\}\;.$$

The stiffness matrix for a homogenized composite can be determined as follows:

(13) $$H_{k}=\frac{H_{e}^{T}\;K\;H_{e}}{area}\;.$$

The described homogenization method turns the full 3D model into a simplified shell model, which allows for shortening the duration of the computations while maintaining high accuracy of the results.

The matrix $H_{k}$ is composed of matrices **A**, **B**, **D,** and **R** according to the following equation:

(14) $$H_{k}=\left[\begin{array}{ccc} A_{3\times 3} & B_{3\times 3} & 0\\ B_{3\times 3} & D_{3\times 3} & 0\\ 0 & 0 & R_{2\times 2} \end{array}\right]\;,$$

where **A** contains tensile and shear stiffnesses, **B** contains coupling of tensile and bending stiffnesses, **D** contains bending and torsional stiffnesses, and **R** contains transverse shear stiffness.

In cases of symmetrical cross-sections, the matrix **B** is the zero matrix. However, if the cross-section is asymmetric, non-zero terms appear in matrix **B**, which results from the coupling between bending/torsional curvatures and tensile/shear forces, and affects the values in the matrix **D**. Traditionally, this problem was solved by minimizing matrix **B** with an appropriate selection of the neutral axis. The uncoupled matrix **D** can be computed using the following equation:

(15) $$D=D^{\prime }-BA^{-1}B\;,$$

where $D^{\prime }$ contains bending and torsional stiffnesses for non-zero matrix **B**.

### 2.3. Edge Crush Test

**Figure 3:**
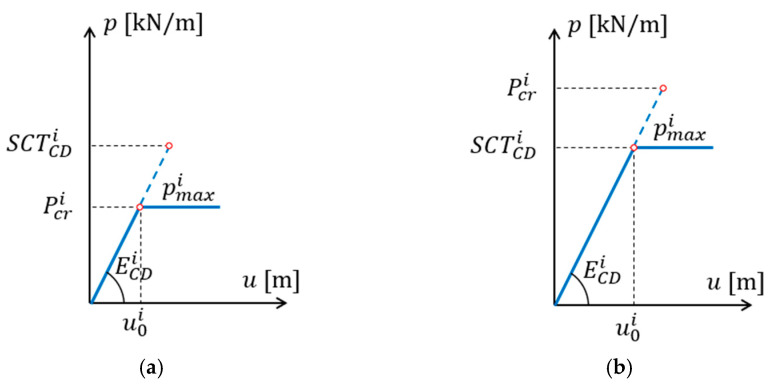
Maximum load of the *i*-th layer: (**a**) the case where the critical load is lower than the compressive strength; (**b**) the case where the compressive strength is lower than the critical load.

For the analytical determination of the BCT value, it is necessary to know the ECT value, which can be obtained by summing the strength of all layers, including the take-up factor:

(16) $$\mathrm{ECT}=\overset{n}{\underset{i=1}{\sum }}\;p_{max}^{i}\alpha_{i}\;,$$

where $\alpha_{i}$ is the take-up factor (see Table 2) and $p_{max}^{i}$ is the maximum load of the i-th layer. The value of this load can be the compressive strength $SCT_{CD}{}^{i}$ or critical load $P_{cr}^{i}$, whichever occurs first (see Figure 3):

(17) $$p_{max}^{i}=\min\left(SCT_{CD}^{i},\;P_{cr}^{i}\right)\;.$$

The critical load can be computed in many ways. An overview of the formulae was presented by Garbowski et al. [14]. For the determination of ECT, the critical load for rectangular orthotropic panels was calculated from the following:

(18) $$P_{cr}^{L}=\frac{1}{\alpha^{2}}\left[D_{11}\alpha^{4}+2\left(D_{12}+2D_{33}\right)\alpha^{2}\beta^{2}+D_{22}\beta^{4}\right]\;,$$

where:

(19) $$\alpha =\frac{m\pi }{H}\;,\;\;\;\;\;\;\;\;\;\;\beta =\frac{\pi }{L}\;,$$

(20) $$D_{11}=\frac{1}{w}E_{1}I\;,\;\;\;\;\;\;\;\;\;\;D_{22}=\frac{1}{w}E_{2}I\;,$$

(21) $$D_{12}=\frac{\nu_{21}}{w}E_{1}I=\frac{\nu_{12}}{w}E_{2}I\;,\;\;\;\;\;\;\;\;\;\;D_{33}=G_{12}I\;,$$

(22) $$I=\frac{t^{3}}{12}\;,\;\;\;\;\;\;\;\;\;\;w=1-\nu_{12}\nu_{21}\;,$$

where m is the number of half-waves for which $P_{cr}^{L}$ reaches the minimum, $E_{1}$ is the modulus of elasticity in MD, $E_{2}$ is the modulus of elasticity in CD, $\nu_{12}$ and $\nu_{21}$ are Poisson’s coefficients in the plane, $G_{12}$ is the in-plane shear modulus, and t is the thickness of the layer.

### 2.4. Box Compression Test

After determining the ECT value and the critical load, the BCT value can be computed. For a rectangular package, the compressive strength can be obtained from the formula [14]:

(23) $$\mathrm{BCT}={\mathrm{ECT}}^{0.75}\left[\gamma_{L}{\left(P_{cr}^{L}\right)}^{0.25}L+\gamma_{B}{\left(P_{cr}^{B}\right)}^{0.25}B\right]\;,$$

where $P_{cr}^{L}$ and $P_{cr}^{B}$ are the critical loads of the packaging walls, and $\gamma_{L}$ and $\gamma_{B}$ are the reduction coefficients, which can be computed from the following:

(24) $$\gamma_{L}=\sqrt{\frac{L}{B}}\;,\;\;\;\;\;\gamma_{B}=1\;,\;\;\;\mathrm{if}\;L\leq B\;\gamma_{L}=1\;,\;\;\;\;\;\gamma_{B}=\sqrt{\frac{B}{L}}\;,\;\;\;\mathrm{if}\;L>B.$$

The critical loads can be evaluated from Equation (18), but can also be obtained from the formula in which the transverse shear stiffness is also included:

(25) $$P_{cr}^{L}=\frac{1}{\alpha^{2}}\frac{M}{N}\;,$$

where:

(26) $$M=D_{11}\alpha^{4}+2\left(D_{12}+2D_{33}\right)\alpha^{2}\beta^{2}+D_{22}\beta^{4}+\left(\frac{\alpha^{2}}{R_{44}}+\frac{\beta^{2}}{R_{55}}\right)c_{1}\;,$$

(27) $$N=1+\frac{c_{1}}{R_{44}R_{55}}+\frac{c_{2}}{R_{55}}+\frac{c_{3}}{R_{44}}\;,$$

(28) $$c_{1}=c_{2}c_{3}-c_{4}{}^{2}>0\;,$$

(29) $$c_{2}=D_{11}\alpha^{2}+D_{33}\beta^{2}\;,$$

(30) $$c_{3}=D_{33}\alpha^{2}+D_{22}\beta^{2}\;,$$

(31) $$c_{4}=\left(D_{12}+D_{33}\right)\alpha \beta \;.$$

This approach is crucial when the corrugated cardboard is relatively thick (especially for B and C flutes, and double-walled cardboards), and its transverse shear modulus is low, e.g., due to unintentional crushing, during printing, or the lamination process.

### 2.5. The Non-Local Sensitivity Analysis

The non-local sensitivity in this study was carried out for several of the above-described quantities, namely for edge crush resistance (ECT), critical load $\left(P_{cr}\right)$ and box strength to static crushing (BCT). In each case, the parameters of the model are the basis weights of the individual layers of the corrugated board collected in vector x. If by h(x) we denote the quantity whose sensitivity is determined, then through small perturbations, $\Delta x_{i}$ of the i-th layer grammage, a change in the determined quantity $h\left(x\pm e_{i}\Delta x_{i}\right)$ can be computed. Here, $e_{i}$ is a unit vector of i-th grammage in the parameter space. Then, by determining a numerical gradient through, e.g., the central difference, the sensitivity at a specific point in the parameter space (weights of the component papers) can be obtained. Therefore, the sensitivity can be described by the following formula:

(32) $$s=\frac{h\left(x+e_{i}{\Delta x}_{i}\right)-h\left(x-e_{i}\Delta x_{i}\right)}{2\Delta x_{i}}\frac{x_{i}}{h\left(x\right)}\;$$

**Figure 4:**
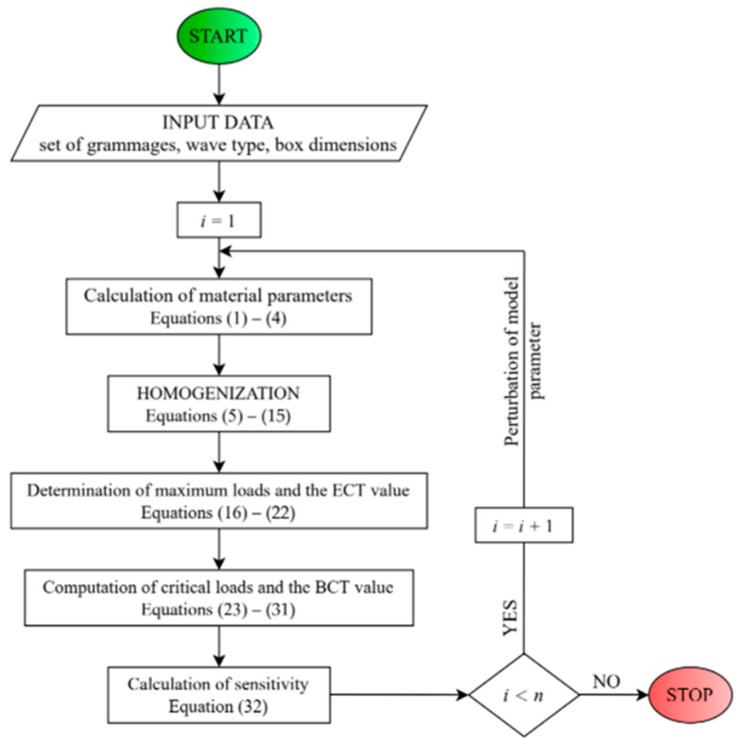
Flowchart of the algorithm for the determination of the non-local sensitivities.

Non-local means here that the sensitivity is checked at many points in the model parameter space in order to build information about the gradients of the studied quantities in the full range of the parameter, and not locally at a specific point in this space. In Figure 4, an algorithm for the determination of non-local sensitivity is shown. In the flowchart, i is the iteration number and n is the number of all perturbing parameters.

## 3. Results

**Figure 5:**
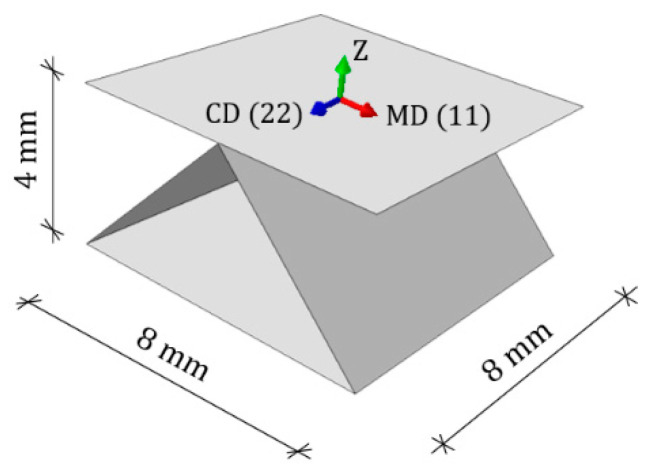
Geometry and main dimensions of the numerical model. Machine Direction (MD), Cross Direction (CD) and thickness direction (Z).

Before proceeding to the sensitivity analysis, which is the main goal of this work, the quality of the homogenization method used here was first checked and validated. For this purpose, in the first step, an analysis of the influence of the number of finite elements on the homogenization result was carried out. Such a check also allows for determining the influence of static condensation on the quality of the solution. The example uses a simple three-layer model with the middle layer in the form of rectilinear sections (zigzag shape, see Figure 5) in order to avoid the effect of discretization of the undulating layer (in which the number of elements affects the exact representation of the waveform).

Figure 5 also shows the main dimensions of the simplified model, while Table 3 summarizes all elastic material parameters and thicknesses of the individual layers used in the calculation model.

Table 4 shows the main stiffnesses obtained by homogenization using various numerical models, ranging from a model composed of only a few finite elements to a model composed of several hundred elements. Table 4 also lists the analytically determined bending stiffnesses $D_{11}$ and $D_{22}$, and the tensile/compressive stiffnesses $A_{11}$ and $A_{22}$. In the case of the direction 11 (MD), only flat layers were included in the analytical calculation of both stiffnesses (assuming that the undulating layer has no influence on the result in this direction). This assumption is true for a sine-shaped corrugated layer, and to a lesser extent for the zig-zag shape of the fluting. However, it was made to simplify the analytical calculations, knowing a-priori that the calculated values will be slightly lower than the real ones (as can be seen in Table 4).

The presented results indicate a good agreement between the analytically calculated stiffnesses and the stiffnesses obtained as a result of homogenization. In addition, the convergence of the numerical models along with the increase in the number of elements is clearly noticeable, which makes it possible to conclude that the homogenization method is correct, and the influence of the static condensation method does not adversely affect the obtained results.

Returning to the main thread of this work, which are sensitivities, all values presented in the following tables and graphs are computed by Equation (32), where h becomes ECT, $P_{cr}$, or BCT. Table 5 presents the sensitivity of ECT computed by Equation (16). The ECT value depends on the SCT in CD, the stiffness in CD and MD (indirectly through a critical load). Thus, ECT becomes the quantity described as a function of the grammage, see, e.g., Equation (1).

The sensitivity of the ECT was computed for the four flutes (B, C, E, and F) and the combinations of the basis weight of the corrugated board layers. To create combinations, the following ranges were adopted: liner grammage from 100 every 20 to 200 $g/m^{2}$ and fluting grammage from 80 every 20 to 160 $g/m^{2}$.

Table 5 shows the specific sensitivity values for the analyzed waves. The second and third columns show the minimum and maximum values that can be obtained with the adopted ranges of the basis weights. The fourth and fifth columns show the average values of sensitivity from all of the analyzed combinations with liner and fluting perturbation.

Table 6 shows the sensitivity of ECT, $P_{cr}$, and BCT depending on the basis weight of the corrugated boards layers (liners and fluting). The presented sensitivities are the averaged values from 120 of different boxes with various dimensions. The smallest dimension of the box base is 100 × 100 and the largest considered dimension is 500 × 300, while the box height varies from 50 to 500.

The results presented here are limited to the B wave only and three indices of corrugated cardboard, i.e., three-ply with a two liners grammage of 100 $g/m^{2}$ and a fluting grammage of 160 $g/m^{2}$, marked as 100-160-100, and two subsequent grades marked as 160-80-160 and 140-100-140.

Table 7 shows the sensitivity of ECT, $P_{cr}$, and BCT depending on the basis weight of the corrugated boards layers (liners and fluting). The presented sensitivities are the averaged values from nine different boxes, which were higher than 400 mm with a base dimension lower than 200.

Table 8 shows the sensitivity of ECT, $P_{cr}$, and BCT depending on the basis weight of the corrugated boards layers (liners and fluting). The presented sensitivities are the averaged values from 36 boxes lower than 150 mm.

**Figure 6:**
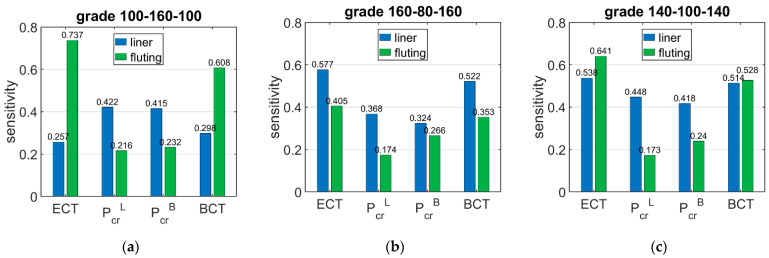
Average sensitivity ECT, *P_cr_*, and BCT of all boxes regarding the liner and fluting perturbation of selected grades: (**a**) 100-160-100, (**b**) 160-80-160, and (**c**) 140-100-140.

Figure 6 shows the sensitivity of ECT, $P_{cr}$, and BCT depending on the basis weight of the corrugated boards layers (liners and fluting). The presented sensitivities are the averaged values from 120 different boxes with various dimensions.

**Figure 7:**
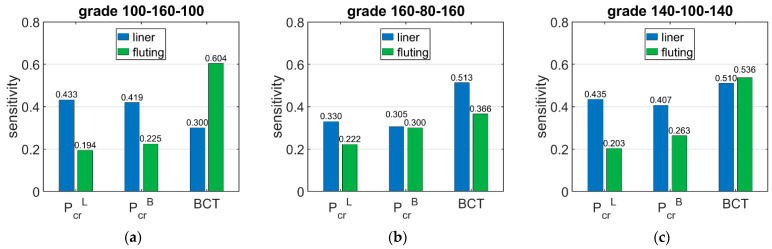
Average sensitivity of *P_cr_* and BCT of high boxes regarding the liner and fluting perturbation of selected grades: (**a**) 100-160-100, (**b**) 160-80-160, and (**c**) 140-100-140.

**Figure 8:**
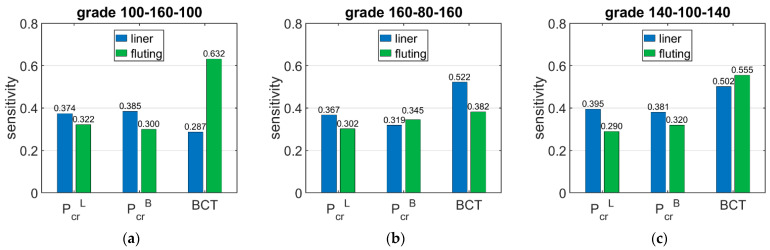
Average sensitivity of *P_cr_* and BCT of stocky boxes regarding the liner and fluting perturbation of selected grades: (**a**) 100-160-100, (**b**) 160-80-160, and (**c**) 140-100-140.

Figure 7 shows the sensitivity of $P_{cr}$ and BCT for high boxes, while Figure 8 shows the sensitivity for stocky boxes. In all cases, just three selected grades were considered and presented, namely: (a) 100-160-100, (b) 160-80-160, and (c) 140-100-140.

**Figure 9:**
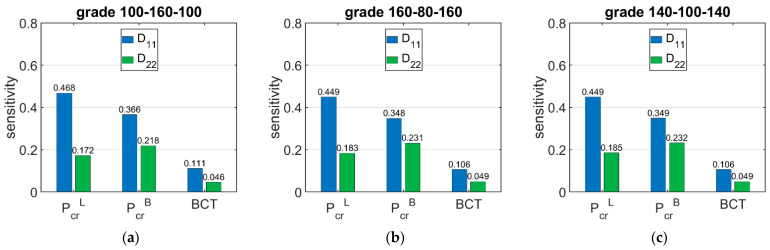
Average sensitivity of *P_cr_* and BCT of all boxes regarding the *D*_11_ and *D*_22_ perturbation of selected grades: (**a**) 100-160-100, (**b**) 160-80-160, and (**c**) 140-100-140.

**Figure 10:**
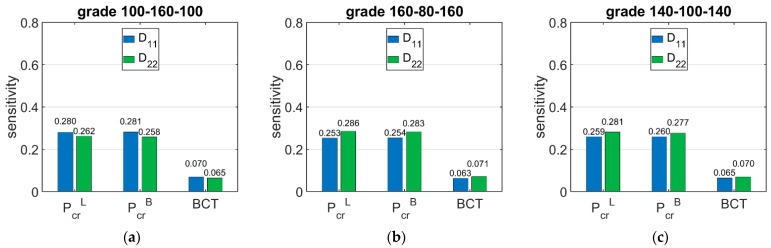
Average sensitivity of *P_cr_* and BCT of high boxes regarding the *D*_11_ and *D*_22_ perturbation of selected grades: (**a**) 100-160-100, (**b**) 160-80-160, and (**c**) 140-100-140.

**Figure 11:**
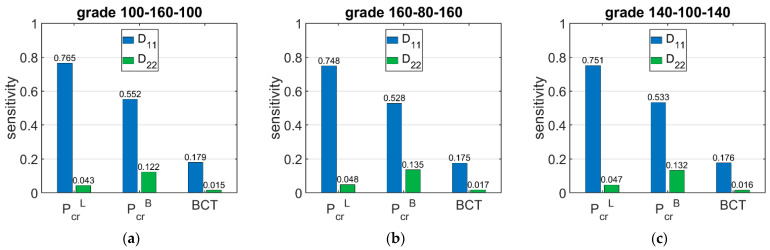
Average sensitivity of *P_cr_* and BCT of stocky boxes regarding the *D*_11_ and *D*_22_ perturbation of selected grades: (**a**) 100-160-100, (**b**) 160-80-160, and (**c**) 140-100-140.

Figure 9 shows the sensitivity of $P_{cr}$ and BCT depending on the bending stiffnesses $D_{11}$ and $D_{22}$. The presented sensitivities are the averaged values from 120 different boxes with various dimensions. Figure 10 shows the sensitivity of $P_{cr}$ and BCT for high boxes, while Figure 11 shows the sensitivity for stocky boxes. In all cases, just three selected grades were considered and presented, namely: (a) 100-160-100, (b) 160-80-160, and (c) 140-100-140.

## 4. Discussion

The conducted analyses allowed for obtaining a complete picture of the sensitivity of both the ECT and the critical load (the main components of the packaging load capacity to static loads), as well as the BCT itself, to small perturbations of the grammage in individual layers of the corrugated cardboard. Table 5 presents the values of the ECT sensitivity to grammage change in the liners and fluting. It is clearly seen that ECT is more sensitive to grammage perturbation for the C wave than for the E wave. At a 10% grammage increase, the minimum sensitivity of ECT is comparable for both wave types (1.9% and 2.0% for the C and E wave, respectively), but the maximum sensitivity of ECT is greater for the C wave (10.9% to 5.2% for the C and E wave, respectively). On average, the ECT sensitivity for the C wave is 53% higher for liner grammage perturbation and 51% higher for fluting grammage perturbation than for the E wave. The main reason is quite obvious—it is because of the distances between the fluting crests and the wave geometry (see Table 2). In the case of the C wave, the wave period and its height are 8 mm and 3.61 mm, respectively, while for the E wave, they are 2.5 mm and 0.76 mm, respectively. Therefore, in the case of the C wave, the loss of stability of both liners and fluting occurs much more often, so that the maximum value of $p_{max}$ in Equation (17) is the critical load and not the SCT value.

Further observations regarding the obtained results of the ECT sensitivity analysis are as follows:

1. As the liner grammage increases, the ECT sensitivity to liner grammage perturbation increases. At the same time, ECT sensitivity to the second liner and fluting grammage perturbation (the grammage of which does not change) decreases.
2. The increase in fluting grammage reduces the ECT sensitivity to liners perturbation.
3. The lowest sensitivity is achieved with low liner grammage perturbation, where the other liner and fluting grammages are high. The greatest sensitivity occurs to the perturbation of the liner with a high grammage with a low grammage for the other liner and fluting.

The results presented in Table 6, Table 7 and Table 8 allow for drawing the conclusions that the sensitivities of the critical load of the longer and shorter walls of the box are similar and range between 3.05% and 4.48% when the basis weight of liner is changed by 10%, while when the basis weight of fluting is changed by 10%, it ranges between 1.73% and 3.00%. The shorter boxes have a slightly higher sensitivity of $P_{cr}$ regarding the grammage of fluting for higher boxes, and varies from 1.94% to 3.00% for high boxes and from 2.90% to 3.45% for stocky boxes.

The situation is slightly different in the case of BCT sensitivity to changes in the grammage of the liners and fluting. The sensitivity of BCT, as for that of the ECT, is dependent on the configuration of the papers in the corrugated board. The sensitivity of BCT to changes in the grammage of liners by 10% ranges from 2.98% to 5.22%, while a change in the grammage of fluting by 10% results in a change in BCT from 3.53% to 6.08%. The difference between the sensitivity of the BCT for low and high boxes is very small and reaches a maximum of 5%.

The results presented in Figure 6, Figure 7 and Figure 8 are the graphical representation of the data compiled in Table 6, Table 7 and Table 8. The results in Figure 9, Figure 10 and Figure 11 exhibit the sensitivity of the critical load and BCT to the change in stiffness. There is a clear trend that the stiffness $D_{11}$ generates higher sensitivities for all quantities than $D_{22}$. The change in $P_{cr}$ resulted from a change in stiffness $D_{11}$ by 10% ranges from 3.48% to 4.49%, while the change in $P_{cr}$ resulted from a change in stiffness $D_{22}$ ranges from 1.72% to 2.32%. The sensitivity of BCT regarding stiffness $D_{11}$ reaches 1.1%, while $D_{22}$ reaches 0.5%. The change of $P_{cr}$ resulted from changes of $D_{11}$ and $D_{22}$ are almost the same in the case of low boxes, while in the case of high boxes, the sensitivity of $P_{cr}$ regarding $D_{22}$ is several times lower than for $D_{11}$.

## 5. Conclusions

Nowadays, it is very important for lightweight material to be used in the production of various structures, including corrugated cardboard packaging. Therefore, understanding and checking the impact of changing the grammage of individual layers of corrugated cardboard on the changes in its mechanical properties is crucial in the optimization process. The paper presents the results of extensive numerical analyzes carried out in order to determine the sensitivity of various quantities for determining the mechanical properties of corrugated cardboard and the packaging that is made of it. The study examined the sensitivity of edge crush resistance (ECT), critical load ($P_{cr}$), and static crushing resistance of packaging (BCT) to changes in the grammage of individual layers of corrugated cardboard. Based on the numerical and computational analyzes carried out here, it is possible to make decisions about changing the composition of the three-layer corrugated cardboard in an easier and more conscious way.

## Figures and Tables

**Table 1:** The Mondi technical data for fluting paper.

Property	Unit of Measure	Grammage (g/m^2^)							
		80	85	90	100	120	130	135	160
$SCT_{CD}$	N/mm	1.36	1.48	1.60	1.80	2.16	2.34	2.43	2.95
${\mathrm{Tensile}\;\mathrm{stiffness}\;\mathrm{index}\;}_{MD}$	Nmm/g	11.0							
${\mathrm{Tensile}\;\mathrm{stiffness}\;\mathrm{index}\;}_{CD}$	Nmm/g	3.8							

**Table 2:** Geometric parameters of waves.

Wave (flute)	Wave Length (mm)	Height (mm)	Take-Up Factor (-)
B	6.5	2.46	1.32
C	8	3.61	1.43
E	3.5	1.15	1.27
F	2.5	0.76	1.25

**Table 3:** Thicknesses and material parameters of individual layers in the numerical model used for validation of the homogenization method.

Layer	Thickness	$E_{1}$	$E_{2}$	$\nu_{12}$	$G_{12}$	$G_{13}$	$G_{23}$
	(mm)	(N/mm^2^)	(N/mm^2^)	(-)	(N/mm^2^)	(N/mm^2^)	(N/mm^2^)
Liner	0.18	9333	3889	0.189	2332	170	111
Fluting	0.16	6875	2375	0.172	1564	125	68
Liner	0.18	9333	3889	0.189	2332	170	111

**Table 4:** The selected stiffnesses computed by various numerical model (with different number of four-node bi-linear FEs used) and simple analytical formula.

Model	Number of Elements	$A_{11}(N/\mathrm{mm})$	$A_{22}(N/\mathrm{mm})$	$D_{11}(\mathrm{Nmm})$	$D_{22}(\mathrm{Nmm})$
FEM 1	8	3608.8	1961.4	13,786	6651.4
FEM 2	32	3565.0	1960.8	13,774	6471.3
FEM 3	112	3553.4	1960.6	13,755	6426.5
FEM-4	448	3549.4	1960.5	13,749	6415.2
FEM-5	1792	3548.0	1960.5	13,746	6412.4
Analytical	-	3360.0	1937.4	13,449	6226.7

**Table 5:** The sensitivity of ECT regarding the grammage of liner or fluting.

Wave (Flute)	Minimum	Maximum	Liner ^1^	Fluting ^1^
B	0.21	0.79	0.42	0.37
C	0.19	1.09	0.52	0.58
E	0.2	0.52	0.34	0.35
F	0.2	0.51	0.34	0.34

^1^ Average value of all grammage cases.

**Table 6:** The sensitivity of ECT, *P_cr_*, and BCT regarding the grammage of liner or fluting.

Grammage	Perturbed Layer	ECT	$P_{cr}^{L}$	$P_{cr}^{B}$	BCT
100-160-100	liner	0.257	0.422	0.415	0.298
	fluting	0.737	0.216	0.232	0.608
160-80-160	liner	0.577	0.368	0.324	0.522
	fluting	0.405	0.174	0.266	0.353
140-100-140	liner	0.538	0.448	0.418	0.514
	fluting	0.641	0.173	0.240	0.528

**Table 7:** The sensitivity of ECT, *P_cr_*, and BCT of high packages (*H* ≥ 400, *L* ≤ 200) regarding the grammage of liners and fluting.

Grammage	Perturbed Layer	ECT	$P_{cr}^{L}$	$P_{cr}^{B}$	BCT
100-160-100	liner	0.257	0.433	0.419	0.300
	fluting	0.737	0.194	0.225	0.604
160-80-160	liner	0.577	0.330	0.305	0.513
	fluting	0.405	0.222	0.300	0.366
140-100-140	liner	0.538	0.435	0.407	0.510
	fluting	0.641	0.203	0.263	0.536

**Table 8:** Sensitivity of ECT, *P_cr_*, and BCT of stocky packages (*H* ≤ 150) regarding the grammage of liners and fluting.

Grammage	Perturbed Layer	ECT	$P_{cr}^{L}$	$P_{cr}^{B}$	BCT
100-160-100	liner	0.257	0.374	0.385	0.287
	fluting	0.737	0.322	0.300	0.632
160-80-160	liner	0.577	0.367	0.319	0.522
	fluting	0.405	0.302	0.345	0.382
140-100-140	liner	0.538	0.395	0.381	0.502
	fluting	0.641	0.290	0.320	0.555

## Data Availability

The data presented in this study are available on request from the corresponding author.
